# Development and validation of the intention to use the ICD-11 questionnaire in the Malaysian medical records context

**DOI:** 10.1371/journal.pone.0308403

**Published:** 2024-09-06

**Authors:** Erwyn Chin Wei Ooi, Zaleha Md Isa, Mohd Rizal Abdul Manaf, Ahmad Soufi Ahmad Fuad, Azman Ahmad, Mimi Nurakmal Mustapa, Nuraidah Mohd Marzuki, Cik Noor Baayah Abdul Jalil, Catherina William Totu

**Affiliations:** 1 Department of Public Health Medicine, Faculty of Medicine, National University of Malaysia, Kuala Lumpur, Malaysia; 2 Health Informatics Centre, Planning Division, Ministry of Health Malaysia, Putrajaya, Malaysia; 3 Medical Record Department, Queen Elizabeth Hospital, Kota Kinabalu, Sabah, Malaysia; Zahedan University of Medical Sciences, ISLAMIC REPUBLIC OF IRAN

## Abstract

As health systems transition to ICD-11, it is essential to gauge the readiness and improve existing transition efforts. Assessing the intention to use ICD-11 and factors influencing it is imperative to encourage the use of ICD-11 among the medical record officers (MROs) and assistant medical record officers (AMROs). This study aims to develop and validate a questionnaire on the factors influencing the intention to use ICD-11 among MROs and AMROs in the Ministry of Health, Malaysia. This study comprised a questionnaire development and validation involving 292 participants nationwide from Ministry of Health Malaysia facilities. The questionnaire was developed based on items adapted from the literature. Forward and backward English-Malay translation was done. Then, the questionnaire was examined for content validity, internal consistency reliability, construct validity, face validity, convergent validity, discriminant validity and confirmatory factor analyses. The final version of the questionnaire consists of eleven domains represented by 50 items. The content validity index and modified kappa were excellent for all domains. The Kaiser-Meyer-Olkin sampling adequacy value was appropriate, with a value of 0.790. The questionnaire also demonstrated good internal consistency reliability with Cronbach’s alpha values between 0.850 and 0.992. Confirmatory factor analysis showed a reasonable fit for this eleven-factor model. In conclusion, this questionnaire provides a reliable tool for investigating the intention to use ICD-11 among MROs and AMROs. Positive findings from the psychometric properties support the validity of the questionnaire. This instrument can potentially support personnel in charge of ICD codification, guide the ICD-11 transition at various levels and facilitate research on support dynamics among the MROs and AMROs.

## Introduction

The International Statistical Classification of Diseases and Related Health Problems (ICD) is a disease classification system that systematically records, interprets, and compares mortality and morbidity statistics collected at different periods and in various nations. ICD allows the codification of the diagnosis of illnesses and other health issues into alphanumeric code, which simplifies data storage, retrieval, and analysis [1]. Essentially, ICD use involves the reporting and codifying of the diagnoses documented by the treating doctors. ICD codification is the clinical coders’ assignment of specific ICD codes to the diagnoses reported by the treating physicians [2]. Current ICD-10 use is a manual process, compared to ICD-11 coding, which is a computerised process to ease the coding experience [3].

In 2019, the 72^nd^ World Health Assembly adopted the ICD-11, which came into effect on Jan 1 2022 [4]. Since then, 64 countries have been in various stages of implementation [5]. For example, pilot implementation activities in Kuwait [6], Iran [7, 8], Rwanda [9] and China [10], whereas information feasibility studies and planning activities have already been done in the USA and Australia respectively [11, 12]. Generally, ICD-11 is a distinct and more efficient system built on formal ontology. It can be used in present IT infrastructures and is adaptable to other classifications and terminology services [13]. The process of using ICD-11 includes the ICD-11 Embedded Classification Tool (ICD-11 ECT). ICD-11 ECT is a search engine developed by the World Health Organization (WHO) to identify suitable ICD-11 codes [3].

From previous experiences, ICD transition has never been a straightforward venture [6, 7, 10]. Up to the latter stages of the change, it is rife with covert difficulties which require adaptations based on local challenges and issues. In other words, there is no one-size-fits-all strategy [7, 14]. The ICD transition’s impact on statistical reporting and reimbursement requires proper planning and firm support from the stakeholders [6]. According to Golpira et al. (2021), conducting feasibility studies in each country is imperative for the ICD-11 transition to succeed [7].

In 2023, the Ministry of Health (MOH), Malaysia has mandated the use of ICD-11 from 2024 onwards at the national level [15–17]. As systems commence transition to ICD-11 in 2024 per the directive, it is critical to learn the factors influencing the intention to use ICD-11 among users in Malaysia as soon as possible [17]. A newly introduced innovation which does not fit the users’ needs will be perceived negatively. Pain points will not be able to be identified and resolved by the policymakers, causing potential users to resist ICD-11 initially. Due to its mandatory implementation, users will be forced to use the system eventually because they do not have the choice [18]. Besides, new technological innovations, like ICD-11, may encounter obstacles due to the technological complexity and the users’ unique characteristics. This is because users transition from manual coding to a computerised process [3]. Previous studies have shown that users of a new technology will reject it if the innovation does not match their attitudes and expectations [19]. Worst, the full potential of ICD-11 may not be realised in Malaysia because users do not intend to use it in the first place. Subsequently, the quality of data collected from healthcare facilities will be negatively impacted. Low-quality data depict inaccurate information on disease trends and the complexity of diseases treated at various levels of facilities in a country [20].

Users have a crucial role in ensuring the successful adoption of ICD-11 since they possess essential knowledge about the factors influencing their adoption decisions [21]. At the Ministry of Health (MOH), Malaysia facilities, ICD usage from codification and reporting is mainly done by the medical record officers (MROs) and the assistant medical record officers (AMROs) [22]. The successful adoption of the ICD-11 depends on the medical records professionals’ intention to use the application [23]. Moreover, measuring the intention to use ICD-11 is more suitable than measuring the usage of the system due to the mandated use of applications like ICD-11 in Malaysia because focusing on the usage will be a hundred per cent [17]. Conversely, intention better reflects the beliefs and motivation of the users towards an innovation like ICD-11 in comparison to innovation usage [24].

With the increasing trend of countries transitioning to ICD-11, including Malaysia, we opine that there is a need for a tool to study and monitor the feasibility of ICD-11 [17]. Several tools have already been used in previous transition-related studies. However, they are mainly focused on assessing the user experience [6], ICD-11 training aspects [25], productivity [8], documentation [26], psychiatry [27], and implementation experience [10]. To the best of our knowledge, no available instruments focus on the intention to use ICD-11 for users in Malaysia. Therefore, an opportunity exists to design and validate an instrument to understand the intention to use ICD-11 and the factors influencing it in the Malaysian context. With that in mind, the current study aims to examine and validate the scale of a model comprising variables influencing the intention to use ICD-11 in the Malaysian setting.

## Literature review

Several well-established theories focus on the psychological constructs influencing the intention to use innovation. For example, the Technology Acceptance Model (TAM) [28], Extended Technology Acceptance Model (TAM2) [29], Unified Theory of Acceptance and Use of Technology (UTAUT) [30], Theory of Planned Behaviour (TPB) [31], and Decomposed Theory of Planned Behaviour (DTPB) [32]. These theories have been adjusted and verified in a variety of situations and contexts [33–37].

TPB can better predict and explain behaviours in a compulsory setting in comparison to TAM [18]. At the same time, the extended versions of TAM, like TAM2 and UTAUT, are similar to TPB [33]. However, the decomposed model has superior explanatory and predictive capacity than TPB. This is because, Decomposed Theory of Planned Behaviour (DTPB) offers more profound insights and a more robust picture of the technology adoption phenomenon [18]. For example, DTPB has been widely applied in the domains of agriculture [34], education [35, 36], banking [37, 38] and healthcare technologies [23, 39]. Therefore, in the context of this study, with ICD-11 as the innovation in focus [13], we will apply the DTPB as a framework for questionnaire design and validation. The study hypothesises that the scale based on DTPB will exhibit a satisfactory factor structure.

### Decomposed theory of planned behaviour (DTPB)

Based on TPB, the DTPB was introduced in 1995 by Taylor and Todd [32]. According to DTPB, an individual’s attitude (ATT), subjective norm (SN) and perceived behavioural control (PBC) contribute to their behavioural intention (INT) [31, 32]. In the DTPB, attitude is further decomposed into perceived ease of use (PEOU), perceived usefulness (PU) and compatibility (COM), subjective norm into interpersonal (II) and external influences (EI) and perceived behavioural control into facilitating conditions (FC) and self-efficacy (SE) [32].

Intention (INT) is the envisioned outcome that directs a person’s planned actions like using ICD-11. An MRO or AMRO’s intents reveal what could spur their actions in using ICD-11 in a particular way and the related motivations. As a result, intention should be anticipated to impact and influence performance to the degree that the MRO or AMRO possesses behavioural control [40]. Concerning the questionnaire design and DTPB, intention to use ICD-11 is influenced by ATT, SN and PBC [32].

In this study’s context, attitude (ATT) is the degree to which a person has a positive or negative assessment or appraisal of using ICD-11 [41]. In other words, a user’s convictions and characteristics define their attitude. On the use of Electronic Health Records, it has been shown that user’s positive attitude towards a newly introduced technology will improve the chances of the technology being used in the future [21].

Subjective norms (SN) are the social pressures the MROs and AMROs feel. In general, the pressures are from their surroundings to either use ICD-11 or not, as well as the influence that supervisors and colleagues may have on the person’s decision to use ICD-11 [31]. Prior research has demonstrated that respondents are more motivated to carry out the desired behaviour when these social groups significantly impact them [39]. For example, in the use of electronic medical records exchange among physicians, subjective norms are a critical factor influencing doctors’ inclinations to use information systems in their practice [39].

Perceived behavioural control (PBC) is defined as how easy or difficult ICD-11 is viewed to be performed by the MROs and AMROs [31]. PBC considers medical records professionals’ previous experiences and anticipated challenges affecting their confidence in utilising ICD-11 [40]. Innovation adoption among healthcare workers has shown that if the user has the resources and the appropriate guidance, the staff will likely have positive intentions to use the new system [42].

### Decomposition of attitude

This study’s three constructs influence attitude: perceived usefulness, perceived ease of use, and compatibility. Perceived usefulness is defined as the degree to which the MROs and AMROs believe that ICD-11 will be able to improve their work performance [43]. Previous studies on innovation acceptance have shown that perceived usefulness predicts attitude toward technology [23, 44]. Specifically in the healthcare context involving healthcare consumers on EHRs, Mathai et al. (2022) have found that users’ attitudes toward a technological system like EHRs are positively impacted by their perceptions that these tools offer them definite benefits [21].

Another significant predictor of attitude toward technological innovation is perceived ease of use. Within the scope of this study, perceived ease of use is defined as the degree of medical records professionals’ belief that ICD-11 is easy to use [32]. Previous studies on using health technology systems have shown an empirical relationship between perceived ease of use and the staff’s attitude. Regardless of the level of education among healthcare professionals, if the technology is not easy to use, users will form an unfavourable attitude towards the technology [23, 32].

The compatibility construct defines the degree to which the MROs and AMROs opine that ICD-11 fulfils the current needs and fits their values [45]. As the agent of change, the MOH must be aware of the users’ needs about ICD-11. With this awareness, MOH can recommend improvements to fulfil the identified needs [46]. This is because adoption happens more quickly when the user believes ICD-11 is compatible with their workflows and values [47].

### Decomposition of subjective norm

Two determinants influence the subjective norms in this study: external influence and interpersonal influence [32]. Prior studies have shown that the individual’s social group may strongly influence behaviour, especially when using innovation [48]. Peers, colleagues, and immediate supervisors often form the core of the MROs or AMROs social group, influencing users at the interpersonal level. Important information is communicated frequently in this group of ICD-11 users (50). Studies focusing on the use of electronic brokerages showed that adopters may give more weight to the first-hand accounts from peers or superiors, thereby influencing the subjective norm [49].

Besides interpersonal influence, the user’s external influence affects the subjective norms of the user. This study defines external influences from the WHO, specialists and the MOH [49]. In other words, external influence is not personal or specific to the user. In any new initiatives involving new systems, incentives offered to the users affect the subjective norms of the user in a positive way [39]. In Malaysia, the MOH, with close cooperation from the WHO, has actively organised activities and discussions for regular updates and new information. Besides that, the MOH organised awareness and training sessions to inform and as a platform for the local stakeholders to share their views [50].

### Decomposition of perceived behavioural control

Consistent with DTPB and this study, the users’ perceived behavioural control is influenced by the facilitating conditions and their self-efficacy in ICD-11 use [32]. Facilitating conditions among medical records professionals is defined as the extent to which they think the infrastructure and facilities are there to support the use of ICD-11 [30]. For example, ICD-11 ECT supports the ICD-11 code search [3, 13]. Related to the technology used in the healthcare industry, Mathai et al. (2022) found a significant relationship between facilitating conditions and the consumers’ perceived behavioural control [21].

Previous studies have shown that self-efficacy is an additional factor influencing users’ perceived behavioural control [32]. In this study, self-efficacy is an assessment of the MROs and AMROs ability to do what is necessary and to handle potential scenarios related to ICD-11 [51]. Users’ self-efficacy level will influence their choices, readiness, and effort in ensuring the success of ICD-11 implementation. For example, in a study by Hung et al. (2011) involving doctors, self-efficacy directly and significantly impacted the doctors’ perceived behavioural control towards using the Medline system [23].

## Materials and methods

### Study design

This is a questionnaire development and validation study on the factors influencing the intention to use ICD-11 among MROs and AMROs involved in ICD-11 coding. This study consists of two stages: Questionnaire design phase (Phase I) and psychometric testing phase (Phase II).

### Ethics approval

The Research Ethics Committee, Universiti Kebangsaan Malaysia (UKM PPI/111/8/JEP-2023-080) and the Medical Research & Ethics Committee, Ministry of Health (MOH) Malaysia (NMRR ID-23-00756-KIH (IIR) approved this study.

### Phase I: Questionnaire design

#### Item development

The theoretical model DTPB guided the instrument development. We adapted items from prior studies to the context of this study into 12 parts, where 11 sections consisted of 11 psychological constructs (INT, AT, SN, PBC, PU, PEOU, CO, II, EI, SE, FC). The remaining section consists of questions to capture demographic data. Fifty-eight measurement items (Table 1) were considered for the development of construct measures.

#### Content validity

The items then underwent a content validity process by five expert panels to examine the draft questionnaire for relevance, clarity, simplicity, and ambiguity [52]. They comprised three Public Health specialists registered with the National Specialist Register, one Information Technology officer with at least five years of experience and one educator with at least five years of experience in questionnaire development [53].

Several criteria exist for selecting instrument reviewers. Some listed characteristics include documented experience, professional certification, and the ability to present and publish professional papers or initiate research. Given the complexity of the study, experts from specific disciplines can be content reviewers for data collection instruments [54]. To the best of our knowledge, there is no set rule for a minimum number of years of experience to be defined as an expert in content validity exercise. However, based on similar studies conducted in contexts like education [55, 56] and healthcare [53, 57, 58], we have decided on the criteria for experts’ selection to having at least five years of experience in Public Health or questionnaire development or information management. Efforts have been made to ensure that the chosen experts have fulfilled most of the abovementioned criteria.

We then calculated the content validity index (CVI) after the panels scored each scaled item based on relevance, clarity, ambiguity, and simplicity [52]. The panellists also provided opinions or thoughts on the items by completing the questionnaire’s comment section. We improved the draft questionnaire based on the expert panel’s recommendations. Items with I-CVI of ≥0.78 were retained, items with I-CVI of 0.70–0.78 were amended, and items with I-CVI of ≤0.70 were removed [59]. We also computed modified kappa (K), probability of change agreement (P_c_), scale-level content validity index based on universal agreement method (S-CVI/UA), and scale-level content validity index based on average techniques (S-CVI/Ave).

#### Face validity

We pretested the draft questionnaire on five participants who fulfilled the inclusion criteria and provided their consent. The objective of this face validity was to get input from a convenient sample of participants and to ascertain how well the participants understood the items so that it was free of ambiguities. The participants answered the questionnaire and gave their viewpoints. The questionnaire underwent a minor modification based on the feedback [60].

#### Translation

On the language of the questionnaire, we decided to provide a dual language questionnaire rather than one in each language so that respondents could comprehend the questions on a combined level. Even though Malay is the country’s official language, English is a common second language in Malaysia. These two languages are frequently used interchangeably in Malaysia [61]. We used the forward-backward translation method to translate the measurement items from English to Malay. A Malay translator and a Medical Officer fluent in English and Malay forward translated the English version into Malay. Subsequently, two independent translators with full English proficiency translated the Malay version back to English. The study team then harmonised the two translations into a single document.

### Phase II: Psychometric testing of questionnaire

#### Study population

MROs and AMROs employed by the Ministry of Health, Malaysia (N = 479), were the population of this study. We exclude external personnel from non-MOH facilities sent for attachment at MOH facilities, non-medical records professionals involved in the ICD-11 use process and staff who have just returned from long leaves in the past year [62, 63].

#### Sampling method, data collection and sample size estimates

A convenience sampling method was undertaken. Participants were recruited from the ICD-11 training and awareness sessions organised by the MOH. The participants then completed the questionnaire using the provided Google Form link. Personnel who agreed to participate consented online (by checking the "I agree" box) before answering the survey. No information on participants’ identifiers was collected, and participants did not need to log in to their accounts to access the Google Form.

To calculate the appropriate sample size for the survey, we utilised the G*Power 3.1.9 power analysis tool. From the tool, at least 77 responses were needed based on parameters such as effect size of 0.15, α at 0.05 and power at 0.80 [64]. However, evaluating the sample size for EFA, it is advised that samples should be at least 100 [65]. Moreover, a sample size of 160–300 valid observations was recommended for structural equation modelling (SEM), accounting for the target population. For instance, in this study’s context for CFA, a sample size of 200 is deemed large for a population of 400 [66].

#### Study tool

The final 50-item questionnaire in both Malay and English consisted of two main sections: ’Intention to use ICD-11’ (6 items), ’Attitude’ (4 items), ’Subjective norm’ (4 items), ’Perceived behavioural control’ (4 items), ’Perceived ease of use’ (6 items), ’Perceived usefulness’ (5 items), ’Compatibility’ (3 items), ’External influence’ (3 items), ’Interpersonal influence’ (4 items), ’Self-efficacy’ (3 items), ’Facilitating conditions’ (8 items). In addition, study participants’ sociodemographic characteristics were collected. A 7-point Likert scale was used to explore all questionnaire items (Strongly agree/ Extremely important; Agree/ Important; Slightly agree/ Slightly important; Neutral; Slightly disagree/ Slightly unimportant; Disagree/ Unimportant; Strongly disagree/ Extremely unimportant). The finalised survey items are shown in the S1 Appendix.

#### Exploratory factor analysis

Cronbach’s α coefficient was used for internal consistency reliability assessment, and the proposed domain structure and its substructure were analysed using exploratory factor analysis (EFA). Bartlett’s sphericity test and the Kaiser-Meyer-Olkin (KMO) were used to measure sampling adequacy. The dataset was deemed sufficient for factor analysis if the KMO value exceeds 0.50 and Bartlett’s sphericity test result is p<0.05 [67]. Varimax rotation was utilised for the principal component factor analysis. Ideal values were 0.55 or higher, and items with values lower than 0.55 will be dropped from this study [68]. We used Cronbach’s α coefficient to measure the items’ internal consistency reliability (IC). Items with Cronbach’s α coefficient value of ≥0.70 are deemed satisfactory internal consistency reliability [69].

#### Confirmatory factor analysis

This study used confirmatory factor analysis (CFA) to validate the best-fitting factor model among the MRO and AMRO at MOH facilities. CFA was done in first order. A chi-square to the degree of freedom ratio (χ2/df) value of 2.0 with a p-value >0.05, composite fit index (CFI) of 0.95, root mean square error of approximation (RMSEA) of <0.08 were among the criteria used to choose the best-fit model. The determination of the fit indices is based on Hair et al. (2018), that is, using at least one index for absolute fit measure and one incremental fit index [65]. CFA is also utilised to evaluate the constructs’ validity, including the draft questionnaire’s convergent and discriminant validity. Convergent validity is when components that make up a construct converge or share significant variation. The amount that the connected latent construct accounts for of the variation in the corresponding item is shown by the square of the standardised regression weights (SRW). Therefore, to calculate convergent validity, the average variance extract (AVE) formula is as follows:

$$AVE=\frac{\sum_{i=1}^{n}\lambda_{i}^{2}}{n}$$

Factor loadings are represented by λ. A latent construct explains more than 50% of a construct’s variation if each construct’s AVE is more than 0.50. On the other hand, discriminant validity quantifies how well a construct captures a phenomenon not explained by other constructs in the model. Therefore, to assess discriminant validity, we compared whether the AVE of any concept in the model is higher than the square of the correlation between any two constructs [70]. The software used was SPSS Statistics version 27.0 (IBM Corp., Armonk, NY, USA) and Amos 28.0 programs (IBM Corp., Armonk, NY, USA) for data analysis.

## Results

### Phase I: Questionnaire design

#### Items of the constructs of decomposed theory of planned behaviour (DTPB)

Table 1.

**Table 1:** Measures of the constructs of Decomposed Theory of Planned Behaviour (DTPB).

Psychological Construct	Items	Descriptions
**Intention to Use (INT)** [42]	INT1	I am willing to spend time to learn about ICD-11.
	INT2	I am willing to get involved in ICD-11 use related activities.
	INT3	I am willing to improve the professional authority of the Medical Record profession by leveraging various opportunities to meet the demand for ICD-11 use.
	INT4	I am willing to take advantage of various opportunities to gain knowledge to meet the demand for ICD-11 specialised Medical Record personnel.
	INT5	I am willing to take advantage of various opportunities to gain knowledge on the application of ICD-11 to meet the demand for ICD-11 specialised Medical Record personnel.
	INT6	I have actively attended on-the-job training courses on ICD-11.
	INT7	I have attended on-the-job training courses organised internally at the workplace.
**Attitude (ATT)** [23]	ATT1	In my opinion, I. . . with the idea of coding in ICD-11.
	ATT2	Using ICD-11 is pleasant.
	ATT3	It is useful to use ICD-11.
	ATT4	In general, I … with the coding of diagnoses according to ICD-11.
**Subjective Norms (SN)** [23]	SN1	Most people who are important to me (colleagues) would think that I should learn about ICD-11.
	SN2	Most people who are important to me (colleagues) would think that I should use ICD-11 at workplace.
	SN3	The people who influence (superiors) my decisions would think I should learn ICD-11.
	SN4	The people who influence (superiors) my decisions would think I should use ICD-11 at workplace.
**Perceived Behavioural Control (PBC)** [23]	PBC1	I have the resources, knowledge and ability to learn about ICD-11.
	PBC2	I have the resources, knowledge and ability to use ICD-11.
	PBC3	I would be able to learn the methods of using ICD-11.
	PBC4	I would be able to apply the methods of using ICD-11.
**Perceived Usefulness (PU)** [23]	PU1	I find that ICD-11 is useful in my work.
	PU2	Using ICD-11 in my work will help me to complete tasks faster.
	PU3	Using ICD-11 will improve my work performance.
	PU4	Using ICD-11 in my work will increase my productivity.
	PU5	Using ICD-11 will increase my work effectiveness.
	PU6	Using ICD-11 makes my work easy.
**Perceived Ease of Use (PEOU)** [23]	PEOU1	Reference materials on ICD-11 are clear and easy to understand.
	PEOU2	I will find it easy to learn to use ICD-11.
	PEOU3	I will find it easy to find the correct ICD-11 codes.
	PEOU4	I will find it easy to become proficient using ICD-11.
	PEOU5	I find that ICD-11 is flexible in terms of use.
	PEOU6	I find that ICD-11 is easy to use
**Compatibility (COM)** [71]	COM1	The use of ICD-11 is compatible with the way I work.
	COM2	The use of ICD-11 is compatible with my working practices.
	COM3	The use of ICD-11 meets the needs of my work.
**Interpersonal Influence (II)** [49]	II1	My friends and colleagues think that I should use ICD-11.
	II2	My acquaintances consider that the use of ICD-11 is a good idea.
	II3	My acquaintances have influenced me to try using ICD-11 in clinical coding.
**External Influence (EI)** [32]	EI1	I have read MOH and WHO publications which stated that using ICD-11 is a good way to perform clinical coding.
	EI2	MOH and/or WHO demonstrate(s) positive sentiment towards the use of ICD-11.
	EI3	MOH and/or WHO have published materials that influenced me to try ICD-11 coding.
**Self-efficacy (SE)** [23]	SE1	I have sufficient knowledge to do ICD-11 coding on my own.
	SE2	I will not feel awkward to use ICD-11 by myself.
	SE3	I am capable to use ICD-11 independently.
	SE4	The ability to independently code with ICD-11 comfortably is …
	SE5	The ability to use ICD-11 without anyone’s assistance is. . .
	SE6	Being knowledgeable enough to code in ICD-11 is…
	SE7	Being comfortable coding ICD-11 on my own is…
	SE8	Being able to code in ICD-11 even if no one is around to tell me how to use it is. . .
**Facilitating Conditions (FC)** [23]	FC1	I can use ICD-11 at any time that I want to
	FC2	I have prior coding skills that can help with ICD-11 coding.
	FC3	I make an effort to use ICD-11.
	FC4	I have enough time needed to familiarise myself with using ICD-11.
	FC5	I have sufficient time to code in ICD-11.
	FC6	I have access to necessary resources (computer) required to use ICD-11.
	FC7	I have access to necessary resources (internet) required to use ICD-11.
	FC8	Having sufficient time to use ICD-11 is. . .
	FC9	Being able to have the time needed to get used to ICD-11 is. . .
	FC10	Being able to have the time to use this ICD-11 is. . .

#### Translation and adaptation

*Forward translation*. Harmonisation of the English to Malay translations from two translators resulted in the final version of the questionnaire. The researchers and the translators discussed the differences until they reached a consensus on the suitability of the words or terms used.

*Backward translation*. For backward translation, two translators proficient in Malay and English independently translated the completed Malay questionnaire back into English. The researchers and the translators discussed the differences until they reached a consensus on the suitability of the words or terms used. Most items have meanings identical to those of the original English questionnaire.

#### Content validation

Five expert panels assessed the draft questionnaire’s content validity for intention, attitude, subjective norms, perceived behavioural control, perceived usefulness, perceived ease of use, self-efficacy, facilitating conditions, compatibility, and interpersonal and external factor domains. For each of the domains, the following indicators of content validity are as follows: (1) Content validity index (I-CVI); (2) Scale-level content validity index based on average methods (S-CVI/AVE); (3) Scale-level content validity index based on universal agreement method (S-CVI/UA); (4) Probability of change agreement (P_c_) and; (5) modified kappa (K) based on relevance, clarity, simplicity and ambiguity (Table 2).

**Table 2:** Evaluation of I-CVI and modified kappa (k*).

Item	Panel in agreement	I-CVI	UA	P_c_	Kappa	Interpretation	S-CVI/ Ave	S-CVI/ UA
INT1	5	1.00	1	0.03	1.00	Excellent	1.00	1.00
INT2	5	1.00	1	0.03	1.00	Excellent		
INT3	5	1.00	1	0.03	1.00	Excellent		
INT4	5	1.00	1	0.03	1.00	Excellent		
INT5	5	1.00	1	0.03	1.00	Excellent		
INT6	5	1.00	1	0.03	1.00	Excellent		
INT7	5	1.00	1	0.03	1.00	Excellent		
ATT1	5	1.00	1	0.03	1.00	Excellent	1.00	1.00
ATT2	5	1.00	1	0.03	1.00	Excellent		
ATT3	5	1.00	1	0.03	1.00	Excellent		
ATT4	5	1.00	1	0.03	1.00	Excellent		
SN1	5	1.00	1	0.03	1.00	Excellent	1.00	1.00
SN2	5	1.00	1	0.03	1.00	Excellent		
SN3	5	1.00	1	0.03	1.00	Excellent		
SN4	5	1.00	1	0.03	1.00	Excellent		
PBC1	5	1.00	1	0.03	1.00	Excellent	1.00	1.00
PBC2	5	1.00	1	0.03	1.00	Excellent		
PBC3	5	1.00	1	0.03	1.00	Excellent		
PBC4	5	1.00	1	0.03	1.00	Excellent		
PU1	5	1.00	1	0.03	1.00	Excellent	1.00	1.00
PU2	5	1.00	1	0.03	1.00	Excellent		
PU3	5	1.00	1	0.03	1.00	Excellent		
PU4	5	1.00	1	0.03	1.00	Excellent		
PU5	5	1.00	1	0.03	1.00	Excellent		
PU6	5	1.00	1	0.03	1.00	Excellent		
PEOU1	5	1.00	1	0.03	1.00	Excellent	1.00	1.00
PEOU2	5	1.00	1	0.03	1.00	Excellent		
PEOU3	5	1.00	1	0.03	1.00	Excellent		
PEOU4	5	1.00	1	0.03	1.00	Excellent		
PEOU5	5	1.00	1	0.03	1.00	Excellent		
PEOU6	5	1.00	1	0.03	1.00	Excellent		
COM1	5	1.00	1	0.03	1.00	Excellent	1.00	1.00
COM2	5	1.00	1	0.03	1.00	Excellent		
COM3	5	1.00	1	0.03	1.00	Excellent		
II1	5	1.00	1	0.03	1.00	Excellent	1.00	1.00
II2	5	1.00	1	0.03	1.00	Excellent		
II3	5	1.00	1	0.03	1.00	Excellent		
EI1	5	1.00	1	0.03	1.00	Excellent	1.00	1.00
EI2	5	1.00	1	0.03	1.00	Excellent		
EI3	5	1.00	1	0.03	1.00	Excellent		
SE1	5	1.00	1	0.03	1.00	Excellent	1.00	1.00
SE2	5	1.00	1	0.03	1.00	Excellent		
SE3	5	1.00	1	0.03	1.00	Excellent		
SE4	5	1.00	1	0.03	1.00	Excellent		
SE5	5	1.00	1	0.03	1.00	Excellent		
SE6	5	1.00	1	0.03	1.00	Excellent		
SE7	5	1.00	1	0.03	1.00	Excellent		
SE8	5	1.00	1	0.03	1.00	Excellent		
FC1	5	1.00	1	0.03	1.00	Excellent	1.00	1.00
FC2	5	1.00	1	0.03	1.00	Excellent		
FC3	5	1.00	1	0.03	1.00	Excellent		
FC4	5	1.00	1	0.03	1.00	Excellent		
FC5	5	1.00	1	0.03	1.00	Excellent		
FC6	5	1.00	1	0.03	1.00	Excellent		
FC7	5	1.00	1	0.03	1.00	Excellent		
FC8	5	1.00	1	0.03	1.00	Excellent		
FC9	5	1.00	1	0.03	1.00	Excellent		
FC10	5	1.00	1	0.03	1.00	Excellent		

No items were deleted based on the expert panel’s comments and the results. However, items II1 and EI1 were deemed to be double-barrelled and restructured. The questionnaire draft, as a result of the comments by the panels, consisted of seven items for the INT domain, four items for the ATT domain, four items for the SN domain, four items for the PBC domain, six items for the PU domain, six items for the PEOU domain, three items for the COM domain, four items for the II domain, four items for the EI domain, eight items for the SE domain and ten items for the FC domain.

#### Face validity

Five participants who fulfilled the inclusion criteria completed the questionnaire draft with all items within ten to fifteen minutes. The finding suggests that the participants thought the questionnaire was straightforward and understandable. The 60 items remained with minor adjustments per recommendation from the participants.

### Phase II: Psychometric testing

#### Sociodemographic characteristics of participants

The population of MROs and AMROs, which shared similar characteristics of the intended sample population for the psychometric assessment, was the subject of this study. A total of 299 participants consented to be involved in the study. One participant did not give consent and seven participants did not complete the questionnaire, leaving 292 valid questionnaires with a response rate of 97.3%. Females comprised the majority (78.8%) and were of Malay ethnicity (81.2%). The respondents’ ages ranged from 23 to 59 (mean 40.2 ± 7.0) years. 49.7% of the respondents had completed at least a high school or diploma. AMROs comprised the majority of respondents (91.1%) with years of experience ranging from 0 to 31 (mean 9.0 ± 6.5) years of experience. The sociodemographic summary of the respondents is tabulated in Table 3.

**Table 3:** Summary distribution of the sociodemographics of the medical record professionals (N = 292).

Factors	Frequency, n	Percentage (%)	Mean (SD)
**Sociodemographic**			
Gender			
Male	62	21.2	
Female	230	78.8	
Age (years)			40.2 (± 7.0)
Ethnicity			
Malay	237	81.2	
Chinese	3	1.0	
Others	52	17.8	
Level of education			
Diploma/STP/STPM/STAM/HSC	145	49.7	
Bachelor’s degree	87	29.8	
Masters	60	20.5	
Grade of appointment			
MRO	23	7.9	
AMRO	269	91.1	
Number of years of ICD coding (years)			9.0 (± 6.5)

#### Construct validity

For construct validity, 105 Medical Record Officers (MRO) and Assistant Medical Record Officers (AMRO) participated in a cross-sectional study. The participants’ average age was (M_age_ = 40.1, SD = 7.99). The participants consisted of 102 (97.14%) Malay, with 84 (80.00%) females and 21 (20.00%) males. Using the EFA, we examined the suitable constructs for all the domains. Bartlett’s sphericity test results were less than 0.001, and the KMO showed 0.785 based on the EFA result, suggesting that the data were appropriate for factor analysis. We adopted items with factor loading greater than 0.50.

With eigenvalues greater than 1, 11 components were found to be contributing to 88.30% of the total variance. The first component explained the variance by 32.68%, the second component by 13.14%, the third component by 8.89%, the fourth component by 7.53%, the fifth component by 5.94%, the sixth component by 5.23%, the seventh component by 3.93%, the eighth component by 3.34%, the ninth component by 2.75%, the tenth component by 2.60% and the eleventh component by 2.27% of the variance, respectively.

Following the varimax rotation (Table 6), we excluded FC9-FC10, INT7, and PU1 items. SE1, SE5-SE8 were excluded from the FC, INT, PU and SE domains. There are 51 items in the updated version of the questionnaire. The extracted factors are as in Table 4:

**Table 4:** Summary of the results of the items, factor loadings, communalities, and eigenvalue for the rotated factors.

Item		Factor Loadings	Variance Explained	Eigenvalue
Factor 1: Facilitating conditions			32.68%	16.665
FC1	I can use ICD-11 at any time that I want to	0.777		
FC2	I have prior coding skills that can help with ICD-11 coding.	0.781		
FC3	I have time to use ICD-11.	0.908		
FC4	I make an effort to use ICD-11.	0.903		
FC5	I have enough time needed to familiarise myself with using ICD-11.	0.878		
FC6	I have sufficient time to code in ICD-11.	0.884		
FC7	I have access to necessary resources (computer) required to use ICD-11.	0.921		
FC8	I have access to necessary resources (internet) required to use ICD-11.	0.904		
FC9 (removed)	Being able to have the time needed to get used to ICD-11 is …	<0.600		
FC10 (removed)	Being able to have the time to use ICD-11 is …	<0.600		
Factor 2: Intention to use ICD-11			13.14%	6.700
INT1	I am willing to spend time to learn about ICD-11.	0.885		
INT2	I am willing to get involved in ICD-11 use related activities.	0.894		
INT3	I am willing to improve the professional authority of the Medical Record profession by leveraging various opportunities to meet the demand for ICD-11 use.	0.904		
INT4	I am willing to take advantage of various opportunities to gain knowledge to meet the demand for ICD-11 specialised Medical Record personnel.	0.905		
INT5	I am willing to take advantage of various opportunities to gain knowledge on the application of ICD-11 to meet the demand for ICD-11 specialised Medical Record personnel.	0.902		
INT6	I have actively attended on-the-job training courses on ICD-11.	0.893		
INT7 (removed)	I have attended on-the-job training courses organised internally at the workplace.	<0.600		
Factor 3: Perceived ease of use			8.89%	4.536
PEOU1	Reference materials on ICD-11 are clear and easy to understand.	0.669		
PEOU2	I will find it easy to learn to use ICD-11.	0.847		
PEOU3	I will find it easy to find the correct ICD-11 codes.	0.824		
PEOU4	I will find it easy to become proficient using ICD-11.	0.811		
PEOU5	I find that ICD-11 is flexible in terms of use.	0.824		
PEOU6	I find that ICD-11 is easy to use	0.854		
Factor 4: Perceived usefulness			7.53%	3.842
PU1 (removed)	I find that ICD-11 is useful in my work.	0.611		
PU2	Using ICD-11 in my work will help me to complete tasks faster.	0.898		
PU3	Using ICD-11 will improve my work performance.	0.926		
PU4	Using ICD-11 in my work will increase my productivity.	0.906		
PU5	Using ICD-11 will increase my work effectiveness.	0.908		
PU6	Using ICD-11 makes my work easy.	0.911		
Factor 5: Attitude			5.94%	3.030
ATT1	In my opinion, I. . . with the idea of coding in ICD-11.	0.828		
ATT2	Using ICD-11 is pleasant.	0.869		
ATT3	It is useful to use ICD-11.	0.892		
ATT4	In general, I … with the coding of diagnoses according to ICD-11.	0.785		
Factor 6: Subjective norms			5.23%	2.666
SN1	Most people who are important to me (colleagues) would think that I should learn about ICD-11.	0.842		
SN2	Most people who are important to me (colleagues) would think that I should use ICD-11 at workplace.	0.842		
SN3	The people who influence (superiors) my decisions would think I should learn ICD-11.	0.800		
SN4	The people who influence (superiors) my decisions would think I should use ICD-11 at workplace.	0.818		
Factor 7: External influence			3.93%	2.004
EI1	I have read MOH publications which stated that using ICD-11 is a good way to perform clinical coding.	0.893		
EI2	I have read WHO publications which stated that using ICD-11 is a good way to perform clinical coding.	0.883		
EI3	MOH and/or WHO demonstrate positive sentiment towards the use of ICD-11.	0.741		
EI4	MOH and/or WHO have published materials that influenced me to try ICD-11 coding.	0.781		
Factor 8: Interpersonal influence			3.34%	1.703
II1	My friends think that I should use ICD-11.	0.778		
II2	My colleagues think that I should use ICD-11.	0.765		
II3	My acquaintances consider that the use of ICD-11 is a good idea.	0.806		
II4	My acquaintances have influenced me to try using ICD-11 in clinical coding.	0.771		
Factor 9: Perceived behavioural control			2.75%	1.401
PBC1	I have the resources, knowledge and ability to learn about ICD-11.	0.807		
PBC2	I have the resources, knowledge and ability to use ICD-11.	0.820		
PBC3	I would be able to learn the methods of using ICD-11.	0.675		
PBC4	I would be able to apply the methods of using ICD-11.	0.719		
Factor 10: Self-efficacy			2.60%	1.327
SE1 (removed)	I have sufficient knowledge to do ICD-11 coding on my own.	<0.600		
SE2	I will not feel awkward to use ICD-11 by myself.	0.817		
SE3	I am capable to use ICD-11 independently.	0.864		
SE4	The ability to independently code with ICD-11 comfortably is …	0.862		
SE5 (removed)	The ability to use ICD-11 without anyone’s assistance is. . .	0.809		
SE6 (removed)	Being knowledgeable enough to use ICD-11 is …	<0.600		
SE7 (removed)	Being comfortable coding ICD-11 on my own is …	0.752		
SE8 (removed)	Being able to code in ICD-11 even if no one is around to tell me how to use it is …	0.772		
Factor 11: Compatibility			2.27%	1.158
COM1	The use of ICD-11 is compatible with the way I work.	0.865		
COM2	The use of ICD-11 is compatible with my working practices.	0.831		
COM3	The use of ICD-11 meets the needs of my work.	0.758		

#### Reliability

We estimated the internal consistency reliability of each factor and the aggregate value using Cronbach’s alpha. Each factor of the Cronbach’s alpha ranged from 0.850 to 0.992 with an overall Cronbach’s alpha of 0.954. As a result, the internal consistency reliability is satisfactory, confirming that the item correlations on the same components were also satisfactory [72]. The internal consistency of the draft questionnaire is summarised in Table 5.

**Table 5:** The internal consistency reliability of the questionnaire.

Domains	Cronbach’s alpha
Facilitating conditions	0.962
Intention to use ICD-11	0.978
Perceived ease of use	0.951
Perceived usefulness	0.992
Subjective norms	0.966
Interpersonal influence	0.937
Attitude	0.977
Perceived behavioural control	0.930
Self-efficacy	0.951
Compatibility	0.850
External influence	0.938
Overall score	0.954

#### Confirmatory factor analysis

For the remaining survey cases (n = 187), a confirmatory factor analysis (CFA) with a maximum likelihood approach was performed, yielding eleven eleven-component structures. The CFA fit indices (CMIN/DF = 1.887, p < 0.001; RMSEA = 0.069; CFI = 0.923) suggest that the measurement model has a reasonably good fit [65]. The factor structure has confirmatory evidence from the indicators mentioned above.

*The convergent and discriminant validity of the questionnaire*. Tables 6 and 7 summarise the results of the discriminant and convergent validity of the questionnaire. For convergent validity, the AVE of each domain is higher than 0.5; thus, the convergent validity of the questionnaire is achieved. The questionnaire’s discriminant validity is attained, as evidenced by the square of the inter-construct correlations being smaller than the AVE of any of the constructs or domains in the study.

**Table 6:** The convergent and discriminant validity of the questionnaire from the best-fit eleven-factor model assessed with confirmatory factor analysis.

Indicator variables	Latent variables	Standardised loading	Square of standardised loading	The sum of squared of standardised loading	Number of indicators	AVE	Composite Reliability
Item 1	FC	0.796	0.634	5.476	8	0.684	0.945
Item 2	FC	0.777	0.604				
Item 3	FC	0.862	0.743				
Item 4	FC	0.905	0.819				
Item 5	FC	0.845	0.714				
Item 6	FC	0.791	0.626				
Item 7	FC	0.821	0.674				
Item 8	FC	0.814	0.663				
Item 1	INT	0.692	0.479	4.412	6	0.735	0.942
Item 2	INT	0.863	0.745				
Item 3	INT	0.975	0.951				
Item 4	INT	0.988	0.976				
Item 5	INT	0.612	0.375				
Item 6	INT	0.942	0.887				
Item 1	PEOU	0.888	0.789	4.919	6	0.820	0.965
Item 2	PEOU	0.943	0.889				
Item 3	PEOU	0.933	0.870				
Item 4	PEOU	0.921	0.848				
Item 5	PEOU	0.859	0.738				
Item 6	PEOU	0.886	0.785				
Item 2	PU	0.932	0.869	4.689	5	0.938	0.987
Item 3	PU	0.960	0.922				
Item 4	PU	0.981	0.962				
Item 5	PU	0.987	0.974				
Item 6	PU	0.981	0.962				
Item 1	ATT	0.936	0.876	3.542	4	0.886	0.969
Item 2	ATT	0.937	0.878				
Item 3	ATT	0.937	0.878				
Item 4	ATT	0.954	0.910				
Item 1	SN	0.939	0.882	3.706	4	0.927	0.981
Item 2	SN	0.962	0.825				
Item 3	SN	0.974	0.949				
Item 4	SN	0.975	0.951				
Item 1	EI	0.952	0.906	2.284	3	0.761	0.904
Item 2	EI	0.940	0.884				
Item 3 (removed)	EI						
Item 4	EI	0.703	0.494				
Item 1	II	0.816	0.666	2.980	4	0.745	0.921
Item 2	II	0.876	0.767				
Item 3	II	0.908	0.824				
Item 4	II	0.850	0.723				
Item 1	PBC	0.961	0.924	3.151	4	0.788	0.936
Item 2	PBC	0.976	0.953				
Item 3	PBC	0.803	0.645				
Item 4	PBC	0.794	0.630				
Item 2	SE	0.828	0.686	2.463	3	0.821	0.932
Item 3	SE	0.980	0.960				
Item 4	SE	0.904	0.817				
Item 1	COM	0.912	0.832	2.447	3	0.816	0.930
Item 2	COM	0.833	0.634				
Item 3	COM	0.960	0.922				

**Table 7:** Discriminant validity index summary.

	FC	INT	PEOU	PU	ATT	SN	EI	II	PBC	SE	COM
FC	**0.827**	-	-	-	-	-	-	-	-	-	-
INT	0.608	**0.857**	-	-	-	-	-	-	-	-	-
PEOU	0.559	0.496	**0.906**	-	-	-	-	-	-	-	-
PU	0.534	0.539	0.627	**0.969**	-	-	-	-	-	-	-
ATT	0.566	0.751	0.541	0.614	**0.941**	-	-	-	-	-	-
SN	0.571	0.665	0.545	0.640	0.746	**0.963**	-	-	-	-	-
EI	0.372	0.367	0.433	0.397	0.421	0.413	**0.872**	-	-	-	-
II	0.497	0.538	0.623	0.652	0.608	0.571	0.536	**0.863**	-	-	-
PBC	0.659	0.587	0.496	0.555	0.624	0.618	0.388	0.476	**0.888**	-	-
SE	0.309	0.265	0.367	0.309	0.290	0.306	0.325	0.250	0.348	**0.906**	-
COM	0.531	0.560	0.583	0.623	0.662	0.576	0.385	0.809	0.510	0.278	**0.903**

## Discussion

To the best of our knowledge, this is one of the early studies detailing the development and validation of a questionnaire to measure the intention of the MROs and AMROs to use ICD-11 in Malaysia. The final questionnaire has 50 items, divided into eleven subscales (FC, INT, PEOU, PU, SN, II, ATT, PBC, SE, COM, and EI). Based on the DTPB theory, we adapted items from the literature. Subsequently, to ensure composite understanding of the questionnaire in Malaysia, we forward and backward translated the questionnaire from English to Malay.

For content and face validity, five expert panels with specialities ranging from Public Health to Questionnaire Development reviewed the items. Ten questions were deemed double-barrelled, and the items were redesigned to ensure each focused on a specific theme. Then, the MROs and AMROs verified the draft items. This approach assured the tool’s content and face validity at the study’s outset, especially regarding language coherence and relevance.

Exploratory and confirmatory factor analysis provided evidence for the construct validity of the questionnaire in evaluating its psychometric qualities. The EFA yielded an eleven-factor model that explained 88.30% of the study’s variance. These factors were then validated using CFA which showed moderately good fit for the instrument. An acceptable fit is defined as having an RMSEA between 0.060 and 0.080; a close fit is described as having a value less than 0.06 [73]. As the incremental fit indicator, the CFI value confirmed the model’s fitness [65]. The questionnaire also has good internal consistency reliability because all eleven subscales have Cronbach alpha values higher than 0.954.

From the eleven-factor model of the questionnaire in CFA, the AVE for each domain of the questionnaire was higher than 0.6, indicating good convergent validity. The square root of AVE of all domains in the questionnaire was higher than the interdomain correlation coefficients, indicating favourable discriminant validity. Given the evidence of the construct validity findings, all the items and domains are valid to measure the factors influencing the intention to use ICD-11 among the MROs and AMROs.

### Theoretical and practical implications

This study has theoretical and practical implications. Theoretically, this study has extended the application of the DTPB model involving ICD-11, a new health information system involving medical records professionals in the Malaysian context [13]. The majority of previous studies have mostly measured clinical utility and reliability measures. The practical implication of this study is that the output of this study, that is, the validated questionnaire, will aid in understanding the MROs and AMROs readiness, confidence, attitude, perceptions, and psychological antecedents for the ICD-11 use. Consequently, the information will guide the MOH in creating focused training programs and anticipate potential showstoppers during the implementation of ICD-11 in Malaysia [50, 74]. The implementation initiatives should be more effective if the variables and determinants of ICD-11 acceptance are understood during the early stages of implementation. Other healthcare systems may also utilise this instrument to find gaps or improve current ICD-11 implementation efforts. However, it is to be noted that although this tool may give the MROs and AMROs a stronger voice in ICD-11 implementation initiatives in Malaysia, it cannot, by itself, meet all the users’ needs. Strong communication and coordination across the health disciplines and external stakeholders are essential to ensure users are provided with a clear and supportive environment to ensure the success of ICD-11 implementation in Malaysia.

### Strength and limitations

Significantly, from the strength of the questionnaire’s validity, this instrument reduces the gap in the body of literature by providing a linguistically and culturally appropriate tool for evaluating the intention to use ICD-11 among the MROs and AMROs. With the availability of relevant measures, health policymakers and academics can assess the support needs of MROs and AMROs involved in the ICD-11 transition efforts.

Notwithstanding the study’s merits and contributions, it is essential to acknowledge a few limitations. Firstly, selection bias may be introduced by the convenient sampling procedure, which could potentially reduce the findings’ generalizability [75]. The external validity of the questionnaire could be improved by using a more representative and varied sample in future studies. Secondly, the instrument has an unequal number of items across components. The instrument should be as small as possible while maintaining the factor structure and psychometric qualities.

## Conclusion

In conclusion, this questionnaire provides a reliable tool for investigating the intention to use ICD-11 among MROs and AMROs. Positive findings from the psychometric properties support the validity of the questionnaire. This instrument can potentially support personnel in charge of ICD codification, guide the ICD-11 transition at various levels and facilitate research on support dynamics among the MROs and AMROs.

## Supporting information

S1 Appendix (PDF)
