# Supplementary material for: Development and validation of the intention to use the ICD-11 questionnaire in the Malaysian medical records context
Source: PLoS One. 2024 Sep 6;19(9):e0308403. doi: 10.1371/journal.pone.0308403 (PMC11379246; doi:10.1371/journal.pone.0308403)
Supplement: S1 Appendix — (PDF) [file pone.0308403.s001.pdf]

LAMPIRAN A

BORANG SOAL SELIDIK

KAJIAN FAKTOR-FAKTOR YANG MEMPENGARUHI NIAT PENGGUNAAN ICD-11 DALAM KALANGAN PEGAWAI TADBIR DAN PENOLONG PEGAWAI TADBIR (REKOD PERUBATAN) DI FASILITI KEMENTERIAN KESIHATAN MALAYSIA

Tujuan edaran soal selidik ini adalah untuk menilai faktor-faktor yang mempengaruhi niat penggunaan ICD-11 dalam kalangan Pegawai Tadbir dan Penolong Pegawai Tadbir (Rekod Perubatan) di Fasiliti KKM. Soal selidik ini mempunyai 4 Bahagian (A, B, C, D). Oleh itu, sukacita dipohon supaya Tuan/ Puan melengkapkan semua butiran seperti yang tertera. Semua maklumat adalah untuk kegunaan kajian ini sahaja.

<table>
<tr><td colspan="4">BAHAGIAN A: MAKLUMAT SOSIODEMOGRAFI RESPONDEN

SECTION A: RESPONDENT'S SOCIODEMOGRAPHIC INFORMATION</td></tr>
<tr><td colspan="4">Sila lengkapkan butiran maklumat yang berkaitan di tempat yang disediakan atau sila tanda '/' sebagai jawapan di kotak jawapan yang disediakan.
(Please fill in the relevant information at the blank space or tick '/' the relevant option in the box)</td></tr>
<tr><td rowspan="2">1</td><td rowspan="2">Jantina
Sex</td><td>Lelaki
Male</td><td></td></tr>
<tr><td>Perempuan
Female</td><td></td></tr>
<tr><td>2</td><td>Umur
Age</td><td colspan="2"></td></tr>
<tr><td rowspan="4">3</td><td rowspan="4">Bangsa
Race</td><td>Melayu
Malay</td><td></td></tr>
<tr><td>Cina
Chinese</td><td></td></tr>
<tr><td>India
Indian</td><td></td></tr>
<tr><td>Others
Lain-lain</td><td></td></tr>
<tr><td rowspan="4">4</td><td rowspan="4">Tahap pendidikan
Level of education</td><td>Diploma/ STP/ STPM/ STAM/ HSC
Diploma/ STP/STPM/ STAM/HSC</td><td></td></tr>
<tr><td>Ijazah Sarjana Muda
Bachelor's degree</td><td></td></tr>
<tr><td>Sarjana
Masters</td><td></td></tr>
<tr><td>Doktor Falsafah
Doctorate</td><td></td></tr>
<tr><td rowspan="8">5</td><td rowspan="8">Gred penjawatan
Grade of appointment</td><td>N44</td><td></td></tr>
<tr><td>N41</td><td></td></tr>
<tr><td>N40</td><td></td></tr>
<tr><td>N36</td><td></td></tr>
<tr><td>N32</td><td></td></tr>
<tr><td>N29</td><td></td></tr>
<tr><td>N26</td><td></td></tr>
<tr><td>N22</td><td></td></tr>
</table>

6	Hospital/ Fasiliti<br>*Hospital/ Facility*		
7	Bilangan tahun pengalaman pengekodan ICD<br>*Number of years of ICD coding*		
8	Adakah anda pernah bercuti >15 hari berturut-turut sejak setahun yang lalu?<br>*Have you taken any long uninterrupted leaves (>15 days) in the past one year?*	Ya<br>*Yes*	
		Tidak<br>*No*	

**BAHAGIAN B: NIAT PENGGUNAAN ICD-11**<br>***SECTION B: INTENTION TO USE ICD-11***
Sila baca setiap pertanyaan dengan teliti dan nyatakan pilihan anda dengan membulatkan angka yang **menggambarkan perasaan anda dengan paling tepat**<br>*Please read each descriptive statement carefully and indicate your choice by circling the appropriate number that is the number* ***that best describes how you feel about the statements.***<br>• Sangat Setuju/ *Strongly Agree*<br>• Sangat Tidak Setuju/ *Strongly Disagree*

**Bil.**	**Niat penggunaan ICD-11 (INT)**<br>***Intention to use ICD-11***	**Sangat Setuju**						**Sangat Tidak Setuju**
**Saya bersedia untuk…**<br>***I am willing to…***								
INT1	Meluangkan masa untuk mempelajari ilmu berkaitan ICD-11.<br><br>*Spend time to learn about ICD-11.*	1	2	3	4	5	6	7
INT2	Melibatkan diri dengan aktiviti berkaitan penggunaan ICD-11.<br><br>*Get involved in ICD-11 use related activities.*	1	2	3	4	5	6	7
INT3	Meningkatkan kewibawaan kerjaya Rekod Perubatan dengan memanfaatkan pelbagai peluang demi memenuhi permintaan dalam penggunaan ICD-11.<br><br>*Improve the professional authority of the Medical Records profession by leveraging various opportunities to meet the demand for ICD-11 use.*	1	2	3	4	5	6	7
INT4	Memanfaatkan pelbagai peluang untuk menimba pengetahuan bagi memenuhi permintaan kakitangan Rekod Perubatan yang berkemahiran dalam ICD-11.<br><br>*Take advantage of various opportunities to gain knowledge to meet the demand for ICD-11 specialised Medical Records personnel.*	1	2	3	4	5	6	7
INT5	Memanfaatkan pelbagai peluang untuk menimba pengetahuan tentang cara penggunaan ICD-11 bagi memenuhi permintaan kakitangan Rekod Perubatan yang berkemahiran dalam ICD-11.<br><br>*Take advantage of various opportunities to gain knowledge on the application of ICD-11 to meet the demand for ICD-11 specialised Medical Records personnel.*	1	2	3	4	5	6	7

INT6	Melibatkan diri secara aktif dalam kursus latihan berkaitan ICD-11 di tempat kerja.<br><br>*Actively attended on-the-job training courses on ICD-11.*	1 2 3 4 5 6 7

**BAHAGIAN C: SIKAP, NORMA SUBJEKTIF, TANGGAPAN KAWALAN TINGKAHLAKU PENGGUNAAN ICD-11, PERSEPSI KEBERGUNAAN, PERSEPSI MUDAH GUNA, EFIKASI KENDIRI, KEADAAN PEMUDAHCARA, KESERASIAN, FAKTOR DALAMAN & FAKTOR LUARAN**<br><br>***SECTION C: ATTITUDE, SUBJECTIVE NORMS, PERCEIVED BEHAVIOURAL CONTROL, PERCEIVED USEFULNESS, PERCEIVED EASE OF USE, SELF-EFFICACY, FACILITATING CONDITIONS, COMPATIBILITY, INTERPERSONAL FACTOR & INTRAPERSONAL FACTOR***
Sila baca setiap pernyataan dengan teliti dan nyatakan pilihan anda dengan membulatkan angka yang **menggambarkan perasaan anda dengan paling tepat**.<br>*Please read each descriptive statement carefully and indicate your choice by circling the appropriate number that is the number **that best describes how you feel about the statements.***<br>• Sangat Setuju/ *Strongly Agree*<br>• Sangat Tidak Setuju/ *Strongly Disagree*

**Bil.**	**Sikap (ATT)**<br>***Attitude***	**Sangat Tidak Setuju**						**Sangat Setuju**
ATT1	Pada pendapat saya, saya … dengan penggunaan ICD-11.<br><br>*In my opinion, I ... with the idea of coding in ICD-11.*	1	2	3	4	5	6	7
ATT2	Pada pendapat saya, saya … dengan peruntukan dana untuk peralihan ke ICD-11.<br><br>*In my opinion, I … with the allocation of funding for ICD-11 transition.*	1	2	3	4	5	6	7
ATT3	Secara amnya, saya … dengan peruntukan dana untuk peralihan ke ICD-11 di hospital KKM.<br><br>*In general, I … the allocation of funding for the transition to ICD-11 in MOH hospitals.*	1	2	3	4	5	6	7
ATT4	Secara amnya, saya … dengan pengekodan diagnosis menurut ICD-11.<br><br>*In general, I … with the coding of diagnoses according to ICD-11.*	1	2	3	4	5	6	7

**Bil.**	**Norma subjektif (SN)**<br>***Subjective norms***	**Sangat Tidak Setuju**						**Sangat Setuju**
**Kebanyakan orang yang penting kepada saya (rakan sekerja) berpendapat bahawa saya sepatutnya…**<br>***Most people who are important to me (colleagues) would think that I should…***								
SN1	Belajar tentang ICD-11.<br><br>*Learn about ICD-11.*	1	2	3	4	5	6	7

SN2	Menggunakan ICD-11 di tempat kerja.<br><br>*Use ICD-11 at workplace.*	1 2 3 4 5 6 7
**Pihak (pegawai atasan) yang mempengaruhi keputusan saya akan berfikir bahawa saya sepatutnya…**<br>***The people who influence (superiors) my decisions would think I should…***		
SN3	Mempelajari ICD-11.<br><br>*Learn ICD-11.*	1 2 3 4 5 6 7
SN4	Menggunakan ICD-11 di tempat kerja.<br><br>*Use ICD-11 at workplace.*	1 2 3 4 5 6 7

Bil.	Tanggapan kawalan tingkah laku (PBC)<br>*Perceived behavioural control*	Sangat Tidak Setuju … Sangat Setuju
**Saya mempunyai sumber, pengetahuan dan kemampuan untuk…**<br>***I have the resources, knowledge and ability to…***		
PBC1	Belajar tentang ICD-11.<br><br>*Learn about ICD-11.*	1 2 3 4 5 6 7
PBC2	Menggunakan ICD-11.<br><br>*Use ICD-11.*	1 2 3 4 5 6 7
**Saya mampu untuk…**<br>***I would be able to…***		
PBC3	Mempelajari kaedah penggunaan ICD-11.<br><br>*Learn the methods of using ICD-11.*	1 2 3 4 5 6 7
PBC4	Mengamalkan kaedah penggunaan ICD-11.<br><br>*Apply the methods of using ICD-11.*	1 2 3 4 5 6 7

Bil.	Persepsi kebergunaan (PU)<br>*Perceived usefulness*	Sangat Setuju … Sangat Tidak Setuju
**Penggunaan ICD-11…**<br>***Using ICD-11…***		
PU2	Dalam pekerjaan saya akan membantu saya menyelesaikan tugasan dengan lebih cepat.<br><br>*In my work will help me to complete tasks faster.*	1 2 3 4 5 6 7
PU3	Akan meningkatkan prestasi pekerjaan saya.	

	Will improve my work performance.	1 2 3 4 5 6 7
PU4	Dalam pekerjaan saya akan meningkatkan produktiviti saya.<br><br>*In my work will increase my productivity.*	1 2 3 4 5 6 7
PU5	Akan meningkatkan keberkesanan pekerjaan saya.<br><br>*Will increase my work effectiveness.*	1 2 3 4 5 6 7
PU6	Memudahkan pekerjaan saya.<br><br>*Makes my work easy.*	1 2 3 4 5 6 7

**Bil.**	**Persepsi mudah guna (PEOU)**<br>***Perceived ease of use***	**Sangat Setuju** **Sangat Tidak Setuju**
PEOU1	Bahan rujukan berkaitan dengan ICD-11 jelas dan mudah difahami.<br><br>*Reference materials on ICD-11 are clear and easy to understand.*	1 2 3 4 5 6 7
**Saya dapat melakukan perkara berikut dengan mudah…**<br>***I will find it easy to…***		
PEOU2	Belajar untuk menggunakan ICD-11.<br><br>*Learn to use ICD-11.*	1 2 3 4 5 6 7
PEOU3	Mencari kod ICD-11 yang tepat.<br><br>*Find the correct ICD-11 codes.*	1 2 3 4 5 6 7
PEOU4	Menjadi mahir menggunakan ICD-11.<br><br>*Become proficient using ICD-11.*	1 2 3 4 5 6 7
**Saya mendapati bahawa ICD-11…**<br>***I find that ICD-11 is…***		
PEOU5	Fleksibel dari segi penggunaan.<br><br>*Flexible in terms of use.*	1 2 3 4 5 6 7
PEOU6	Mudah untuk digunakan.<br><br>*Easy to use.*	1 2 3 4 5 6 7

Bil.	**Keserasian (COM)**<br>***Compatibility***	**Sangat Setuju**						**Sangat Tidak Setuju**
**Penggunaan ICD-11…**<br>***The use of ICD-11…***								
COM1	Sesuai dengan cara saya bekerja.<br><br>*Is compatible with the way I work.*	1	2	3	4	5	6	7
COM2	Serasi dengan amalan pekerjaan saya.<br><br>*Is compatible with my working practices.*	1	2	3	4	5	6	7
COM3	Memenuhi keperluan pekerjaan saya.<br><br>*Meets the needs of my work.*	1	2	3	4	5	6	7

Bil.	**Faktor dalaman (II)**<br>***Interpersonal influence***	**Sangat Setuju**						**Sangat Tidak Setuju**
II1	Rakan-rakan saya berfikir bahawa saya harus menggunakan ICD-11.<br><br>*My friends think that I should use ICD-11.*	1	2	3	4	5	6	7
II2	Rakan-rakan sejawat saya berfikir bahawa saya harus menggunakan ICD-11.<br><br>*My colleagues think that I should use ICD-11.*	1	2	3	4	5	6	7
**Kenalan saya…**<br>***My acquaintances…***								
II3	Beranggapan bahawa penggunaan ICD-11 ialah idea yang bagus.<br><br>*Consider that the use of ICD-11 is a good idea.*	1	2	3	4	5	6	7
II4	Mempengaruhi saya untuk mencuba menggunakan ICD-11.<br><br>*Have influenced me to try using ICD-11 in clinical coding.*	1	2	3	4	5	6	7

Bil.	**Faktor luaran (EI)**<br>***External influence***	**Sangat Setuju**						**Sangat Tidak Setuju**
EI1	Saya pernah membaca terbitan KKM bahawa penggunaan ICD-11 ialah cara yang bagus untuk pengekodan klinikal.<br><br>*I have read MOH publications which stated that using ICD-11 is a good way to perform clinical coding.*	1	2	3	4	5	6	7

EI2	Saya pernah membaca terbitan WHO yang mengatakan bahawa penggunaan ICD-11 ialah cara yang bagus untuk melakukan pengekodan klinikal.<br><br>*I have read WHO publications which stated that using ICD-11 is a good way to perform clinical coding.*	1 2 3 4 5 6 7
**KKM dan/atau WHO…**<br>**MOH and/or WHO…**		
EI4	Telah menerbitkan bahan yang mempengaruhi saya untuk cuba menggunakan ICD-11.<br><br>*Have published materials that influenced me to try ICD-11 coding.*	1 2 3 4 5 6 7

**Bil.**	**Efikasi kendiri (SE)**<br>***Self-efficacy***	**Sangat Setuju** … **Sangat Tidak Setuju**
**Saya…**<br>***I…***		
SE2	Tidak akan berasa kekok menggunakan ICD-11 dengan sendiri.<br><br>*Will not feel awkward to use ICD-11 by myself.*	1 2 3 4 5 6 7
SE3	Mampu melakukan pengekodan ICD-11 tanpa bantuan sesiapa.<br><br>*Am capable to use ICD-11 independently.*	1 2 3 4 5 6 7
		**Sangat Penting** … **Sangat Tidak Penting**
SE4	Kemampuan untuk menggunakan ICD-11 dengan sendiri tanpa rasa kekok adalah…<br><br>*The ability to independently code with ICD-11 comfortably is …*	1 2 3 4 5 6 7

**Bil.**	**Keadaan pemudahcara (FC)**<br>***Facilitating conditions***	**Sangat Tidak Setuju** … **Sangat Setuju**
**Saya…**<br>***I…***		
FC1	Boleh menggunakan ICD-11 pada bila-bila masa saya mahu menggunakannya.<br><br>*Can use ICD-11 at any time that I want to.*	1 2 3 4 5 6 7
FC2	Memiliki kemahiran pengekodan terdahulu yang boleh membantu saya dengan pengekodan ICD-11.	

	Have prior coding skills that can help with ICD-11 coding.	1 2 3 4 5 6 7
FC3	Mempunyai masa untuk menggunakan ICD-11.<br><br>*Have time to use ICD-11.*	1 2 3 4 5 6 7
FC4	Berusaha untuk menggunakan ICD-11.<br><br>*Make an effort to use ICD-11.*	1 2 3 4 5 6 7
FC5	Mempunyai masa yang mencukupi untuk membiasakan diri dengan ICD-11.<br><br>*Have enough time needed to familiarise myself with using ICD-11.*	1 2 3 4 5 6 7
FC6	Mempunyai masa yang mencukupi untuk menggunakan ICD-11.<br><br>*Have sufficient time to code in ICD-11.*	1 2 3 4 5 6 7
FC7	Mempunyai akses kepada sumber (computer) yang diperlukan untuk menggunakan ICD-11.<br><br>*Have access to necessary resources (computer) required to use ICD-11.*	1 2 3 4 5 6 7
FC8	Mempunyai akses kepada sumber (internet) yang diperlukan untuk menggunakan ICD-11.<br><br>*Have access to necessary resources (internet) required to use ICD-11.*	1 2 3 4 5 6 7
